# Tubulointerstitial nephritis and uveitis syndrome with concurrent macular edema caused by granulomatous uveitis

**DOI:** 10.1002/ccr3.3194

**Published:** 2020-08-07

**Authors:** Tomomi Nishi, Hiroshi Tamura, Yusuke Miyashita, Hitoshi Nakazato

**Affiliations:** ^1^ Department of Pediatrics Faculty of Life Sciences Kumamoto University Kumamoto Japan

**Keywords:** children, granulomatous uveitis, tubulointerstitial nephritis and uveitis

## Abstract

It is difficult to differentiate between TINU syndrome and sarcoidosis in young people with renal abnormalities and ocular lesions. Since the spontaneous prognosis of interstitial nephritis is different between the two, it was considered necessary to actively conduct tests for differentiation.

## Graphical abstract

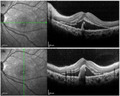

## 1 INTRODUCTION

Tubulointerstitial nephritis and uveitis (TINU) syndrome is complicated with interstitial nephritis of unknown origin. This condition usually presents in adolescent women, and its frequency of occurrence is low. The common form of uveitis associated with TINU is the anterior uveitis, accounting for approximately 80% of cases. Only 20% of cases extend to have intermediate or posterior involvement and most are nongranulomatous uveitis.^1^ Granulomatous uveitis concurrent with TINU syndrome is a rare condition,^2^ as is the co‐occurrence of macular edema.^3^, ^4^

We report the challenges encountered in differentiating granulomatous uveitis with macular edema and present the case of a patient diagnosed with TINU syndrome using renal biopsy.

## 2 CASE

A 13‐year‐old Japanese girl was admitted to a primary ophthalmology department with the chief complaint of blurred vision and declined visual acuity. Her past medical history and her family history were unremarkable. There was no history of any drug use or of extreme fatigue, loss of appetite, or weight loss. She was diagnosed with bilateral granulomatous uveitis. One week later, she was admitted to our hospital with suspected sarcoidosis. Her weight was 50.1 kg (0.1 SD), height was 153.8 cm (−0.4 SD), and her blood pressure was 124/55 mm Hg. She did not have malaise or fever. A physical examination did not reveal any indications of arthritis, stomatitis, or rash.

The patient's visual acuity was 0.4 log MAR in the right eye and 0.5 log MAR in the left eye. Slit‐lamp examination revealed granulomatous keratic precipitates distributed in both eyes. Anterior chamber flare measured by laser flare photometry was 309.5 ± 65.2 photon units/ms in the right eye and 11 385.3 ± 59.4 photon units/ms in the left eye. The intraocular pressure was 10 mm Hg in the right eye and 14 mm Hg in the left eye. There was no vitreous haze, and the fundus was normal in both eyes. Intensive topical corticosteroid therapy was initiated.

The patient's erythrocyte sedimentation rate, C‐reactive protein levels was 0.02 mg/dL, and complete blood count were normal, as were the levels of antinuclear antibodies, anti‐Sjögren's syndrome‐related (SS)‐A antibody, anti‐SS‐B antibody, antiglomerular basement membrane antibody, myeloperoxidase‐antineutrophil cytoplasmic antibody (ANCA), and proteinase 3‐ANCA. Viral infections, such as human T‐cell leukemia virus type 1, cytomegalovirus, herpes simplex virus, vesicular stomatitis virus, hepatitis B virus, and hepatitis C virus, were negative. A QuantiFERON test, performed to rule out tuberculosis as a possible cause of granulomatous uveitis, was also negative, and her chest X‐ray was normal. The levels of angiotensin‐converting enzyme and soluble IL‐2 receptor levels were normal.

The patient's serum creatinine level was 0 0.76 mg/dL, which was mildly elevated, according to her age (normal range for a 13‐year‐old Japanese girl: 0.4‐0.66 mg/dL).^5^ Furthermore, although urinalysis did not indicate proteinuria, her urinary beta 2 microglobulin (β2MG) level was elevated to a creatinine ratio of 2.0 µg/mg creatinine (normal value, <0.35 µg/mg), suggesting renal tubule injury. Consequently, we performed gallium scintigraphy as a whole‐body inflammation evaluation, but no accumulation in the lungs or kidneys was observed. Because the above findings suggested the presence of an inflammatory disease, we suspected sarcoidosis, inflammatory uveitis, or uveitis‐associated collagen diseases. Ultrasonography was then performed to evaluate the patient's kidney function, but there were no indications of renal hypoplasia or hydronephrosis, and the respective renogram revealed no abnormal excretion. The patient's serum creatinine and urinary β2MG level were mildly elevated, and the uveitis was controlled with steroid instillation. Her visual acuity was −0.1 log MAR in the right eye and 0.2 log MAR in the left eye.

**Figure 1:**
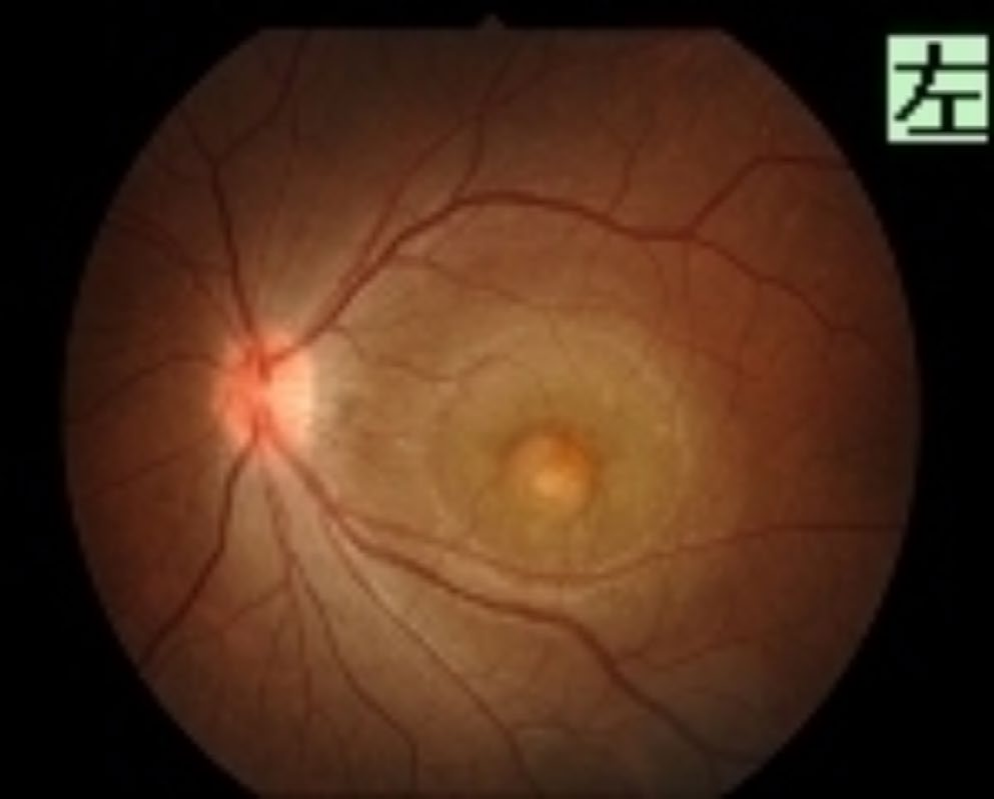
Fundus photograph of the left eye shows a yellow lesion with clear bleeding in the fovea and serous retinal detachment around it

**Figure 2:**
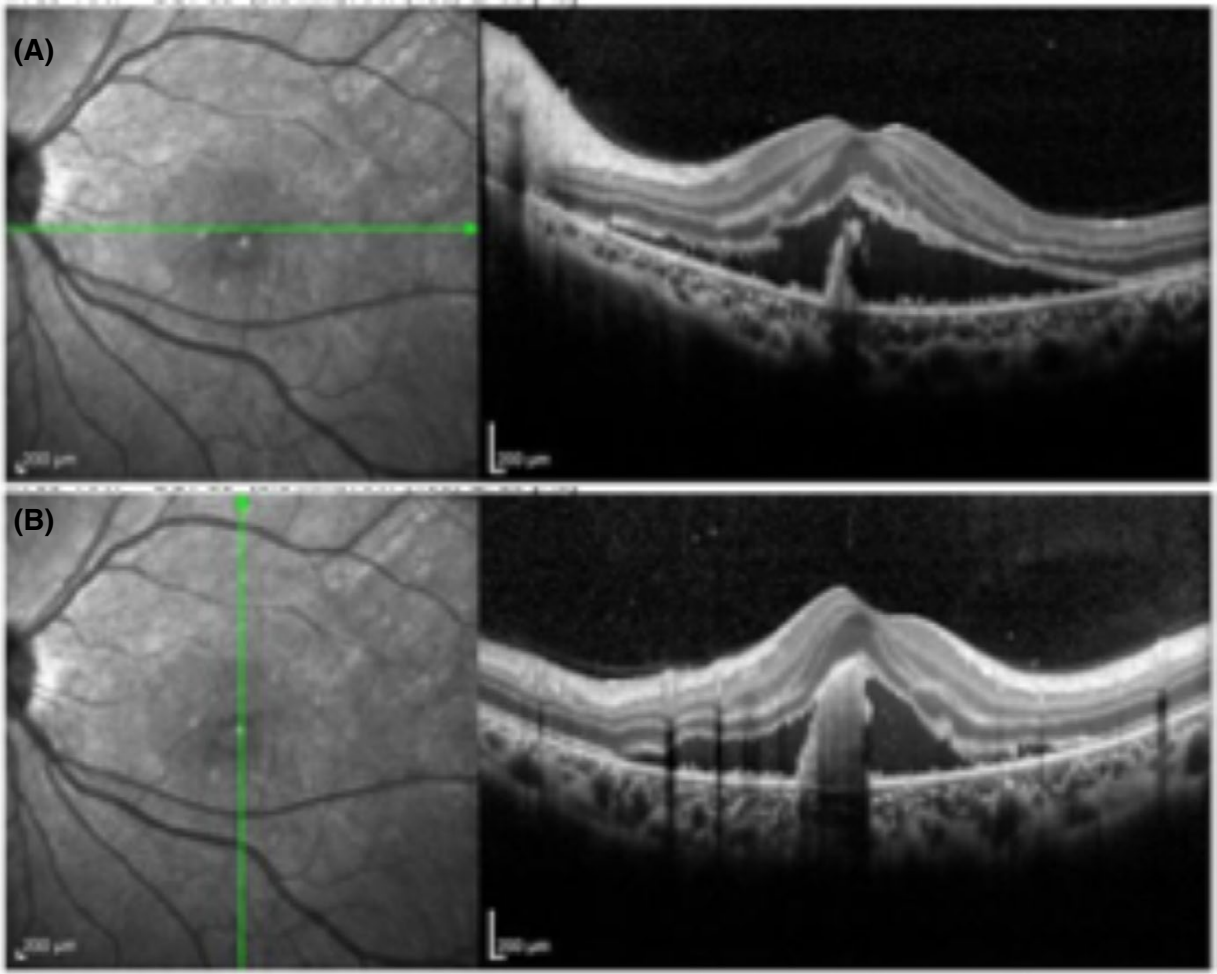
Optical tomographic interferometer with the horizontal line (A) and vertical (B) lines of examination of the left eye shows that the membrane was peeled off and liquid was stored revealing macular edema

**Figure 3:**
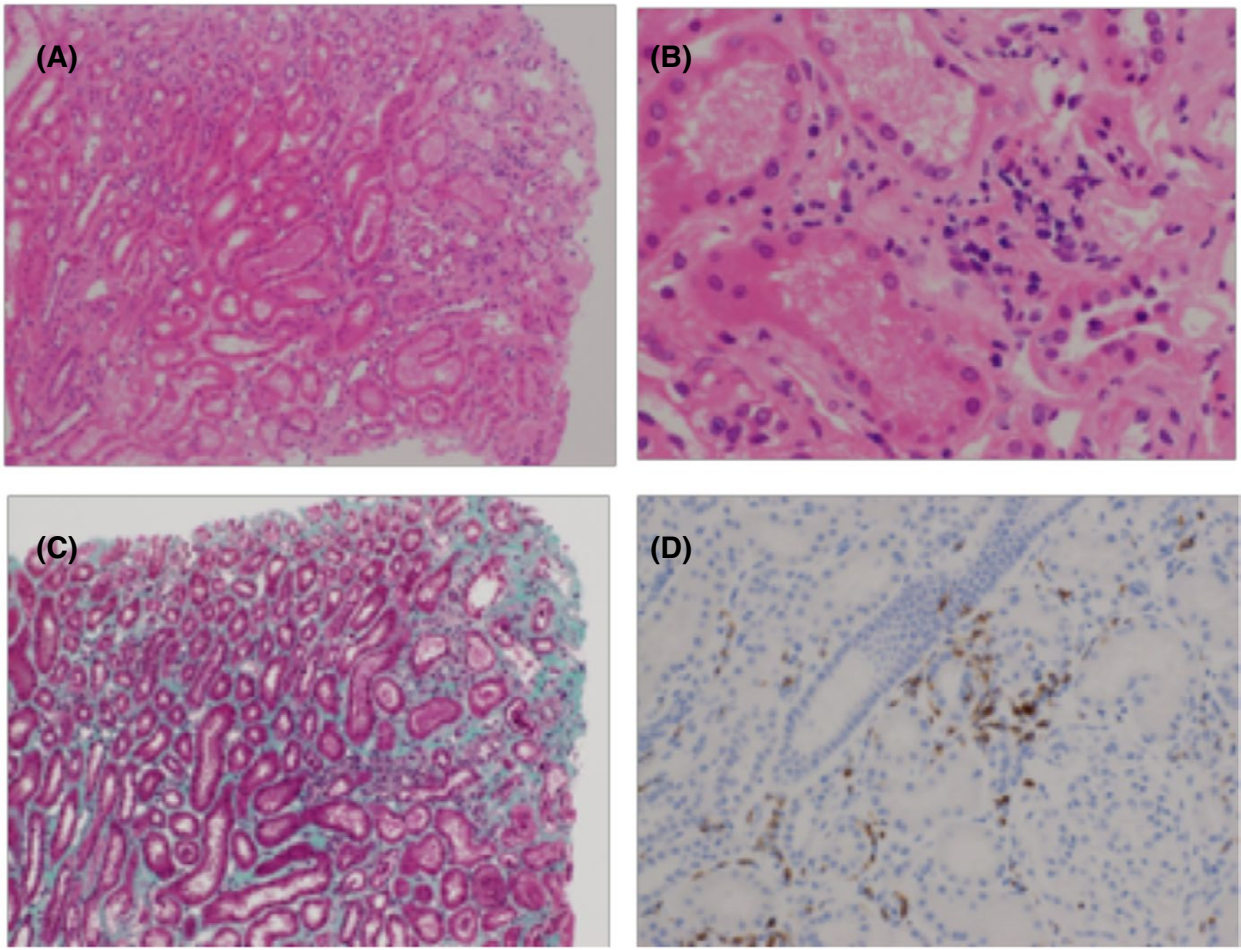
There was no glomerular sclerosis, mesangial proliferative, and membrane change (A,B). Renal stroma has fibrosis (C), and infiltrating blood cells are CD3 positive (D). No granulomatous lesions were observed. A, B: HE staining, C: elastica Masson staining, D: immunostaining (CD 3)

Four months following treatment, scotoma appeared in the center of the patient's left eye, and visual acuity declined 0.7 log MAR. She was diagnosed with a relapse of uveitis, and steroid instillation commenced. However, due to the appearance of macular edema and choroidal neovascularization (CNV), additional treatment for uveitis was required (Figure 1 and Figure 2). The course of uveitis was extremely severe, and we believed that the identification of the original disease was essential for administering additional treatment. A renal biopsy was then performed, which revealed fibrosis of the renal stroma, and the infiltrating blood cells were CD3‐positive. We diagnosed TINU syndrome (Figure 3) and administered oral prednisolone (60 mg/day) to treat the uveitis. Additionally, we administered antivascular endothelial growth factor therapy to treat CNV. The selected course of treatment facilitated mild recovery in her visual acuity. Her visual acuity was 0 log MAR in the left eye. The oral administration of prednisolone was tapered and discontinued within 6 months. Three months after steroid discontinuation, uveitis recurred. Her visual acuity was −0.1 log MAR in the right eye and 0.1 log MAR in the left eye. We administered oral prednisolone (15 mg/d), and the dose was tapered and discontinued within 6 months. Her serum creatinine level and urinary β2MG creatinine ratio were 0.62 mg/dL and 0.25 µg/mg creatinine, respectively, suggesting that her kidney function normalized after prednisone therapy. The uveitis promptly responded to systemic and local corticosteroid treatment, and visual acuity increased to 0 log MAR within 6 days in the left eye. There were no relapses of uveitis or ocular complications during a follow‐up of 2 years.

## 3 DISCUSSION

TINU syndrome is also associated with acute granulomatous uveitis. Therefore, differentiation from collagen diseases such as sarcoidosis, Sjogren's syndrome, and Behcet's disease is often difficult. This patient did not have collagen disease because she tested negative for each autoantibody and there were no major symptoms apart from those of the eyes and kidneys. Behcet's disease was also ruled out because there were no major symptoms other than those of the eyes.

There are more cases with macular edema as a complication of TINU.^6^, ^7^ Macular edema significantly increases the risk of developing vision disorders and visual field disturbances.^8^, ^9^ Gallium scintigraphy failed to reveal any abnormal gallium accumulation. In patients with sarcoidosis, the development of granulomatous lesions in the kidney area and in the eyes ^10^ has been reported, and the presence of these conditions has also been reported in children.^11^ However, pertaining to the patient in this case study, there was no swelling of the hilar lymph nodes and no significant increase in the level of urinary β2MG.^8^ These findings were indicative of atypical sarcoidosis. Additionally, Blau syndrome, a disease that is caused by a mutation in *NOD2* and associated with skin, joint, and ocular symptoms, was also included in the differential diagnosis. Although ocular symptoms usually develop when the medical condition has progressed, there are some cases with preceding ocular symptoms and others where interstitial nephritis has been reported.^12^ Therefore, we performed nucleotide‐binding oligomerization domain‐containing protein 2 analysis, but no abnormality was revealed.

Sarcoidosis was strongly suspected and examined closely, but the diagnostic criteria for sarcoidosis were not satisfied: The kidney biopsy did not show any characteristics of sarcoidosis, no respiratory lesions (hilar lymphadenopathy), and no cardiac abnormalities were found, and characteristic laboratory findings were not detected.

This case was diagnosed as possible TINU syndrome from typical interstitial nephritis and atypical uveitis based on the diagnostic criteria proposed by Mandeville et al.^13^ The renal and ophthalmic prognosis of TINU syndrome are relatively good. However, more than half of TINU patients experience recurrent relapses of uveitis for a long time, and the side effects of steroid treatment are problematic. Hence, it was difficult to employ efficient therapeutic strategies.

Uveitis treatment typically involves steroid instillation or oral administration. In this case, the patient was well treated with oral steroids. However, in some cases, recurrent relapses may develop that cannot be managed with conventional steroid administration. In recent years, cyclosporin, an immunosuppressant used for treating noninfectious uveitis, and adalimumab, a biologic product, have been covered by insurance in Japan.^14^ The treatment of TINU is challenging due to the recurrent course of disease. Thus, immunosuppressants and biologic drugs seem to be a good treatment option because they may prevent the recurrence rate in patients who continue to relapse after treatment with systemic corticosteroids.^7^, ^15^

The benefits are particularly pronounced in young people, reducing the risk of steroid side effect. Adalimumab is used in the management of children and adolescents with JIA‐associated uveitis refractory to MTX. There is robust evidence for it.^16^ Besides, adalimumab was already used the patients with TINU.^7^ 'Typical' uveitis for TINU is described as bilateral acute anterior uveitis presenting within 2 months prior or 12 months after tubulointerstitial nephritis.^17^

We believe that an initial diagnosis of interstitial nephritis should be followed by an eye examination in future clinical practice because uveitis can have a severe course in some cases. Therefore, follow‐up by an ophthalmologist is necessary.

## 4 CONCLUSION

We report a case of granulomatous uveitis complicated with concurrent development of macular edema. The following two characteristics were observed: (a) development of recurrent granulomatous uveitis, even in the presence of TINU syndrome, and (b) mild kidney disorder, even when complicated uveitis was recurrent. It is difficult to differentiate between TINU syndrome and sarcoidosis in young people with renal abnormalities and ocular lesions. Since the spontaneous prognosis of interstitial nephritis is different between the two, it was considered necessary to actively conduct tests for differentiation. In cases with atypical findings, such as this case, even after the diagnosis of TINU syndrome, it is necessary to carefully follow the disease course with the possibility that complications of other diseases, including sarcoidosis, may appear in the future.

## CONFLICT OF INTEREST

None declared.

## AUTHOR CONTRIBUTIONS

TN, YM, and HT: treated the patient. HT and HN: performed the analysis. TN and HT: wrote the paper.

## INFORMED CONSENT

Informed consent was obtained from all individual participants included in the study.

## References

[1] John THM , Ralph DL , Gary NH . The tubulointerstitial nephritis and uveitis syndrome. Survey Ophthalmol. 2001;46(3):195‐208. 10.1016/s0039-6257(01)00261-2 11738428

[2] Kenan B , Turkay R , Nur C , et al. Acute granulomatous iridocyclitis in a child with tubulointerstitial nephritis and uveitis syndrome. J Ophthalmic Inflamm Infect. 2015;5:3. 25861394 10.1186/s12348-015-0035-2 PMC4384971

[3] Hans BH , Daniel C , Manjot KG . Choroidal neovascularization secondary to tubulointerstitial nephritis and uveitis syndrome in an adult patient. Ophthalmic Inflamm Infect. 2015;5:29. 10.1186/s12348-015-0059-7 PMC4596145 26446047

[4] Takemoto Y , Namba K , Mizuuchi K , et al. Two cases of subfoveal choroidal neovascularization with tubulointerstitial nephritis and uveitis syndrome. Eur J Ophthalmol. 2013;23(2):255‐257. 23335312 10.5301/ejo.5000240

[5] Ishikura K , Uemura O , Hamasaki Y , et al. Guidance for evaluating renal function at the time of pediatric chronic kidney disease (pediatric CKD) diagnosis. The Japanese Society for Pediatric Nephrology. 2014;ver1:1‐10.

[6] Mackensen F , Smith JR , Rosenbaum JT . Enhanced recognition, treatment, and prognosis of tubulointerstitial nephritis and uveitis syndrome. Ophthalmology. 2007;114:995‐999. 17383731 10.1016/j.ophtha.2007.01.002

[7] Sobolewska B , Bayyoud T , Deuter C , et al. Long‐term Follow‐up of Patients with Tubulointerstitial Nephritis and Uveitis (TINU) Syndrome. Ocul Immunol Inflamm. 2018;26(4):601‐607. 27937079 10.1080/09273948.2016.1247872

[8] Lardenoye C , Kooji B , Rothova A . Impact of macular edema on visual acuity in uveitis. Ophtahlmology. 2006;113(8):1446‐1449. 10.1016/j.ophtha.2006.03.027 16877081

[9] Taylor SRJ , Lightman SL , Sugar EA , et al. The impact of macular oedema on visual function in intermediate posterior and panuveitis. Ocul Immunol Inflamm. 2012;20(3):171‐181. 22530874 10.3109/09273948.2012.658467 PMC3643807

[10] Masaru A , Suehiro N , Chihiro E , et al. Clinical features of sarcoidosis with renal insufficiency. Japanese J Sarcoidosis. 2012;32(1):101‐105.

[11] Kathryn LP , Deborah LL , Laura SF , et al. Urinary β2‐microglobulin testing in pediatric uveitis: a case report of a 9‐year‐old boy with renal an ocular sarcoidosis. Case Rep Opthalmol. 2015;6:101‐105. 10.1159/000381092 PMC4395824 25873895

[12] Ikuo O , Ryuta N , Nobuo K , et al. Role of *NOD2* genotype in the clinical phenotype of blau syndrome and early‐onset sarcoidosis. Arthritis Rheum. 2009;60(1):242‐250. 19116920 10.1002/art.24134

[13] Marino A , Pagnini I , Giani T , et al. Successful treatment with adalimumab for severe multifocal choroiditis and panuveitis in presumed (early‐onset) ocular sarcoidosis. Int Ophthalmol. 2016;36:129‐135. 26500039 10.1007/s10792-015-0143-x

[14] Zako M . Adalimumab in sarcoidosis patients with refractory non‐infectious uveitis. Japanese J Sarcoidosis. 2017;37:25‐30.

[15] Gion N , Stavrou P , Foster CS . Immunomodulatory therapy for chronic tubulointerstitial nephritis‐ associated uveitis. Am J Ophthalmol. 2000;129:764‐768. 10926986 10.1016/s0002-9394(00)00482-7

[16] Ramanan AV , Dick AD , Jones AP , et al. Adalimumab plus Methotrexate for Uveitis in Juvenile Idiopathic Arthritis. N Engl J Med. 2017;376(17):1637‐1646. 28445659 10.1056/NEJMoa1614160

[17] Mandeville JT , Levinson RD , Holland GN . The tubulointerstitial nephritis and uveitis. Surv Ophthalmol. 2001;46:195‐208. 11738428 10.1016/s0039-6257(01)00261-2

